# COINSTAC: A Privacy Enabled Model and Prototype for Leveraging and Processing Decentralized Brain Imaging Data

**DOI:** 10.3389/fnins.2016.00365

**Published:** 2016-08-19

**Authors:** Sergey M. Plis, Anand D. Sarwate, Dylan Wood, Christopher Dieringer, Drew Landis, Cory Reed, Sandeep R. Panta, Jessica A. Turner, Jody M. Shoemaker, Kim W. Carter, Paul Thompson, Kent Hutchison, Vince D. Calhoun

**Affiliations:** ^1^The Mind Research Network, Lovelace Biomedical and Environmental Research InstituteAlbuquerque, NM, USA; ^2^Department of Electrical and Computer Engineering, Rutgers, The State University of New JerseyPiscataway, NJ, USA; ^3^Department of Psychology and Neuroscience Institute, Georgia State UniversityAtlanta, GA, USA; ^4^Telethon Kids Institute, The University of Western AustraliaSubiaco, WA, Australia; ^5^Departments of Neurology, Psychiatry, Engineering, Radiology, and Pediatrics, Imaging Genetics Center, Enhancing Neuroimaging and Genetics through Meta-Analysis Center for Worldwide Medicine, Imaging, and Genomics, University of Southern CaliforniaMarina del Rey, CA, USA; ^6^Department of Psychology and Neuroscience, University of Colorado BoulderBoulder, CO, USA; ^7^Department of Electrical and Computer Engineering, University of New MexicoAlbuquerque, NM, USA

**Keywords:** decentralized processing, privacy, brain imaging, data sharing, decentralized algorithms

## Abstract

The field of neuroimaging has embraced the need for sharing and collaboration. Data sharing mandates from public funding agencies and major journal publishers have spurred the development of data repositories and neuroinformatics consortia. However, efficient and effective data sharing still faces several hurdles. For example, open data sharing is on the rise but is not suitable for sensitive data that are not easily shared, such as genetics. Current approaches can be cumbersome (such as negotiating multiple data sharing agreements). There are also significant data transfer, organization and computational challenges. Centralized repositories only partially address the issues. We propose a dynamic, decentralized platform for large scale analyses called the Collaborative Informatics and Neuroimaging Suite Toolkit for Anonymous Computation (COINSTAC). The COINSTAC solution can include data missing from central repositories, allows pooling of both open and “closed” repositories by developing privacy-preserving versions of widely-used algorithms, and incorporates the tools within an easy-to-use platform enabling distributed computation. We present an initial prototype system which we demonstrate on two multi-site data sets, without aggregating the data. In addition, by iterating across sites, the COINSTAC model enables meta-analytic solutions to converge to “pooled-data” solutions (i.e., as if the entire data were in hand). More advanced approaches such as feature generation, matrix factorization models, and preprocessing can be incorporated into such a model. In sum, COINSTAC enables access to the many currently unavailable data sets, a user friendly privacy enabled interface for decentralized analysis, and a powerful solution that complements existing data sharing solutions.

## 1. Introduction

While sharing neuroimaging data either prior to or subsequent to a study's completion is becoming more commonplace (Jack et al., 2008; Potkin and Ford, 2009; Poldrack et al., 2013; Eickhoff et al., 2016), two key challenges are emerging. The first involves the volume of data that needs to be processed. In the most widely used computational model (centralized sharing), all shared data are downloaded and processed locally. This entails significant computational and storage requirements, which become barriers to access as data sets increase in size—many groups lack sufficient infrastructure for processing. Secondly, for various reasons, there may be policy restrictions on openly sharing the data. Because neuroimaging has been used as a clinical research tool for nearly two decades, there are many years' worth of studies in which data were collected without the necessary and appropriate consent for *post hoc* data sharing. In some cases the data can be properly anonymized and therefore be categorized as no longer meeting the strict definition of human subjects research, but in other cases institutional or IRB policies may still prevent such data from ever being shared for secondary use. Furthermore, the data may be subject to governmental or proprietary restrictions or there may be prima facie reasons why the data may not ever be de-identifiable. For example, individuals suffering from rare diseases may be identified based solely on their scanning location and disease status (Sweeney, 2002; Sweeney et al., 2015). Thus, in many cases, openly sharing the data may not be possible or feasible (Homer et al., 2008; McGuire et al., 2011). In addition, data sharing is often facilitated by fairly specific data usage agreements (DUA). While DUAs enable sharing, virtually every shared data set has its own DUA, and many of these are approved manually by a committee, so the timeframe to access data that are already shareable can be days, weeks, or even months.

When pooling data for sharing and analysis at a central site is not possible, one approach is to isolate data providers from each other by either locally performing a centrally coordinated analysis or relying on a trusted central computation entity. In both approaches the data is decentralized as it is stored with their respective entities. Keeping the data in their original locations provides a level of control and privacy that is significantly higher than that open sharing. Such an approach also lowers the entry barrier for data providers^1^ (e.g., research or clinical sites). This approach can potentially allow storing and protecting large-scale data within a decentralized schema. Models such as ENIGMA (Thompson et al., 2014, 2015) take this approach, using manual coordination while leaving the data at the site. The goal of this work is to automate access and computation to allow larger-scale analyses on locally-managed data.

### 1.1. Existing alternatives to centralized sharing and their limitations

There are many benefits to enabling automated access to decentralized data. The greatest benefits are the increased statistical power in research studies compared to reliance on available local datasets (Button et al., 2013) and the aforementioned access to otherwise inaccessible data. The recently-proposed Virtual Pooling and Analysis of Research data (ViPAR) approach (Carter et al., 2016) system attempts to capture these benefits. ViPAR uses open-source technologies to isolate data from computation by permanently storing datasets at local sites. To perform an analysis, it temporarily pools subsets of the data via encrypted transfer to a trusted server. ViPAR is an example of work in the right direction: analyses are automated and the data are securely transferred exclusively between the trusted central server and individual providers.

Centralized computation solutions can impose severe bandwidth and traffic requirements, as well as increased computational load. ViPAR pools data to a secure central server for each analysis, distributes the results, and deletes the copies. Multi-band imaging has already led to a 40-fold increase in disk space for functional and diffusion datasets (Feinberg et al., 2010): for truly large-scale neuroimaging studies, repeated data transfers may become challenging for even a small number of sites. In this respect the approach is very similar to the many emerging central data repositories (Insel et al., 2010; Hall et al., 2012; Castellanos et al., 2013; Ivory, 2015)—the data is temporarily centralized each time a computation is performed. Analysis in centralized repositories avoids multiple pooling but the data must be downloaded locally (or to the cloud)^2^.

Centralized computation also does not address privacy issues that prevent data from being transferred directly, even over encrypted links. The enhancing neuroimaging genetics through meta analysis (ENIGMA) consortium (Thompson et al., 2014) takes a pragmatic approach to this issue—they adopt a process that shares summary statistics on locally analyzed data, rather than centralizing the analysis of the original imaging data at a single site. The ENIGMA method has proven very successful (Jack et al., 2008; Thompson et al., 2014; Hibar et al., 2015; Thompson et al., 2015; van Erp et al., 2016). ENIGMA uses both mega- and meta-analysis approaches. In a meta-analysis, which has been used for large-scale genetic association studies, this includes each site performing the same analysis, the same brain measure extraction, or the same regressions; the resulting effect sizes and other statistics are then aggregated. Meta-analyses can summarize findings from tens of thousands of individuals. Since individual-level data are aggregated within a site, the summaries may not be subjected to institutional firewalls or require additional consent forms from subjects (Hibar et al., 2015; van Erp et al., 2016). The overall approach is an incredible leap toward enabling analyses on otherwise inaccessible data.

Although the ENIGMA approach has led to a number of innovative findings through massive international collaborations, there are some challenges that can be addressed by the approach we present in this paper. Firstly, the meta-analyses is effectively executed manually. This is time-consuming: someone has to write the analysis scripts, coordinate with personnel at all participating sites to have the analysis scripts implemented, adapt and debug scripts to ensure correctness at the local sites, and then gather the results back via email or online directories. Second, summary measures are typically restricted to those that can be computed independently of other data. In addition, an analysis using the ENIGMA approach described above is typically “single shot” in that it does not iterate among sites to compute results informed by the data as a whole—this is a meta-analytic solution^3^. From a statistical and machine learning perspective, single-shot model averaging has good asymptotic performance (as the number of subjects and sites becomes very large) (Mcdonald et al., 2009; Zinkevich et al., 2010; Zhang et al., 2012, 2013). However, other work has shown that more communication between sites is needed for statistical consistency (Shamir et al., 2014). In more practical scenarios, more communication can definitely improve performance: simple model averaging does not account for variability between sites due to small sample sizes.

By allowing additional communication, sites can implement a more efficient and accurate learning procedure, especially if the method can be cast as an optimization (Nedic et al., 2000; Nedic and Ozdaglar, 2009; Ram et al., 2010; Duchi et al., 2011). Allowing the computation to touch the data multiple times has many advantages: we show how iterative approaches yield more accurate solutions (closer to the pooled-data mega-analysis) without requiring individual subject measures to be communicated directly.

Iteration also enables the use of many other important methods that cannot be implemented in a single-shot approach^4^. For example, more complex calculations such as feature learning may require iteration and thus not be amenable to a single-shot approach. In particular, simply merging features learned at local sites may not lead to significant gains, but iterated computation and communication between the analyst and the local sites can improve performance. Unlike the big data MapReduce/Hadoop scenarios (e.g., those implemented in Zaharia et al., 2010; Apache Spark, 2015; c.f. H20, 2015 and others) in which the data can be rapidly “shuffled” between iterations, in collaborative research systems the data must stay put and the connections between sites are in many cases heavily bandwidth limited, which leads to different classes of algorithms. In epidemiological research, researchers proposed the dataSHIELD system (Wolfson et al., 2010; Gaye et al., 2014) for decentralized data control. By allowing data holders to maintain control over access, such an approach allows for more privacy protections at the cost of additional labor in implementing and updating a distributed architecture. However, neither the ENIGMA model nor dataSHIELD quantify privacy or provide any guarantees against re-identification (Sarwate et al., 2014) nor does either approach easily enable analyses that require iteration among the sites.

Notably, the sense of protection provided by the plausible (but not quantified) notion of privacy (like in dataSHIELD) may be harmful since there is no way for a user to know if the protection is indeed strong enough. Similarly to dataSHIELD, but deliberately much less technically complex, the Beacon Project (2015) from the Global Alliance for Genomics & Health (GA4GH), provides a simplistic decentralized data sharing or data lookup method for global genomic datasets. The “beacons” provide a web service access point for asking queries in the form of “Do you have a genome with a specific nucleotide at a specific locus?.” While the beacon project has been designed to protect the underlying datasets by returning only allele presence-absence information, recently Shringarpure and Bustamante (2015) have demonstrated that not only is reidentification (of an individual) possible with a small number of queries, but that relatives can also be detected, and that this was possible even with sequencing errors and differences in variant-calling. This susceptibility serves as a cautionary tale when data sharing mechanisms of seemingly privacy protected data can be exploited, and further illustrates the criticality of having multiple levels of privacy protection built into any data sharing methodology.

### 1.2. Requirements for a decentralized model

Ideally, a framework would allow analysis of large scale, but distributed data in a way that (i) provides the same results that would have been obtained if the data were in one place, (ii) preserves the privacy of individual datasets, and (iii) is able to scale to multiple sites and process increasing amounts of data. Cumulatively ViPAR, and ENIGMA (almost) address the first two requirements but not the third. The results produced by an analysis using ViPAR are virtually identical to those that would have been obtained on pooled data, because the data are, indeed, pooled. In ENIGMA the privacy is plausibly preserved by keeping the data at their respective locations and restricting the transferred information to the summary measures only, and thus represents an improved (but not quantified) level of privacy. Nevertheless, ENIGMA supports a message passing infrastructure that could, in principle, implement decentralized computation much more complex than the currently performed “single-shot” meta-analysis. In principle, messages can be passed between sites and the master until convergence to a global analysis. The problem, as with ViPAR, is with scaling. Scaling ViPAR would require powerful central servers of large capacity, while for ENIGMA one would need the large scale coordinated work of many humans. One of the surprising aspects of the ENIGMA approach is that individuals have been so willing to spend their time coordinating such an analysis motivated only by the promise of scientific advancement and publication. We expect a large increase in the willingness to participate if researchers can automate the process of iteration and feedback. In principle, with the addition of dataSHIELD's approach this can be satisfied. However, dataSHIELD currently only fully implements a single method (generalized linear model), in a scripting environment, for relatively low-dimensional data and without an automated way of building new collaborations/consortia within the system. It also does not incorporate preprocessing or feature generation, both of which are important for enabling systems to work with brain imaging data, as we mention later.

### 1.3. Our alternative

In this paper we present our vision of a framework that is designed to address all three goals and we also demonstrate a working prototype system which implements the infrastructure for our proposed solution. We are developing the Collaborative Informatics and Neuroimaging Suite Toolkit for Anonymous Computation (COINSTAC) system that automates federated analyses such as single-shot ENIGMA analyses and other similar systems. Our framework includes iterative approaches, provides ways to run decentralized-data algorithms^5^ on the complete data, and, as an option, a way to impose mathematical privacy guarantees on all shared pieces of information. With COINSTAC we hope to leverage and accelerate community effort as demonstrated in ENIGMA by providing tools that automate such coordination. The use of COINSTAC also has the potential to reduce cost by avoiding the need for powerful centralized servers and reducing the amount of effort needed to coordinate large scale analyses by making them easy to set up and to modify and rerun. A list of challenges and the ways COINSTAC addresses them is summarized in Table 1. As we show, automation opens up the possibility of implementing decentralized analyses that result in the same solutions as pooled-data analyses would. This is possible by automatically iterating among sites with feedback from the central analysis into the next step of the distributed computations. This is not feasible in a system based on human implementation of every step of the analysis but, depending on the algorithm, can either improve results relative to the “single-shot” analysis or be virtually indistinguishable from the pooled-data analysis (Baker et al., 2015). In addition, it also enables the incorporation of more formalized and quantified privacy models, such as differential privacy (Dwork et al., 2006; Dwork and Roth, 2014). In a nutshell, COINSTAC will effectively unify the respective strengths of ViPAR, ENIGMA and dataSHIELD in a single system that is easy for an end user to install and use.

**Table 1:** **The challenges addressed by COINSTAC**.

**Current challenges to research**	**COINSTAC solutions**
Privacy protection for subjects	Differential privacy models
Data sharing concerns for investigators	Raw data are not shared; they are used and summaries are shared
Complex processing streams	A platform for automated, iterated, distributed algorithms
Centralized compute resources	Local computation of input data, centralized aggregation
Quality assurance/data heterogeneity	Anomaly detection and validated quality control methods (planned)
Ease of use	Passive on investigator's part; data reuse occurs without interruption

## 2. The COINSTAC approach

**Figure 1:**
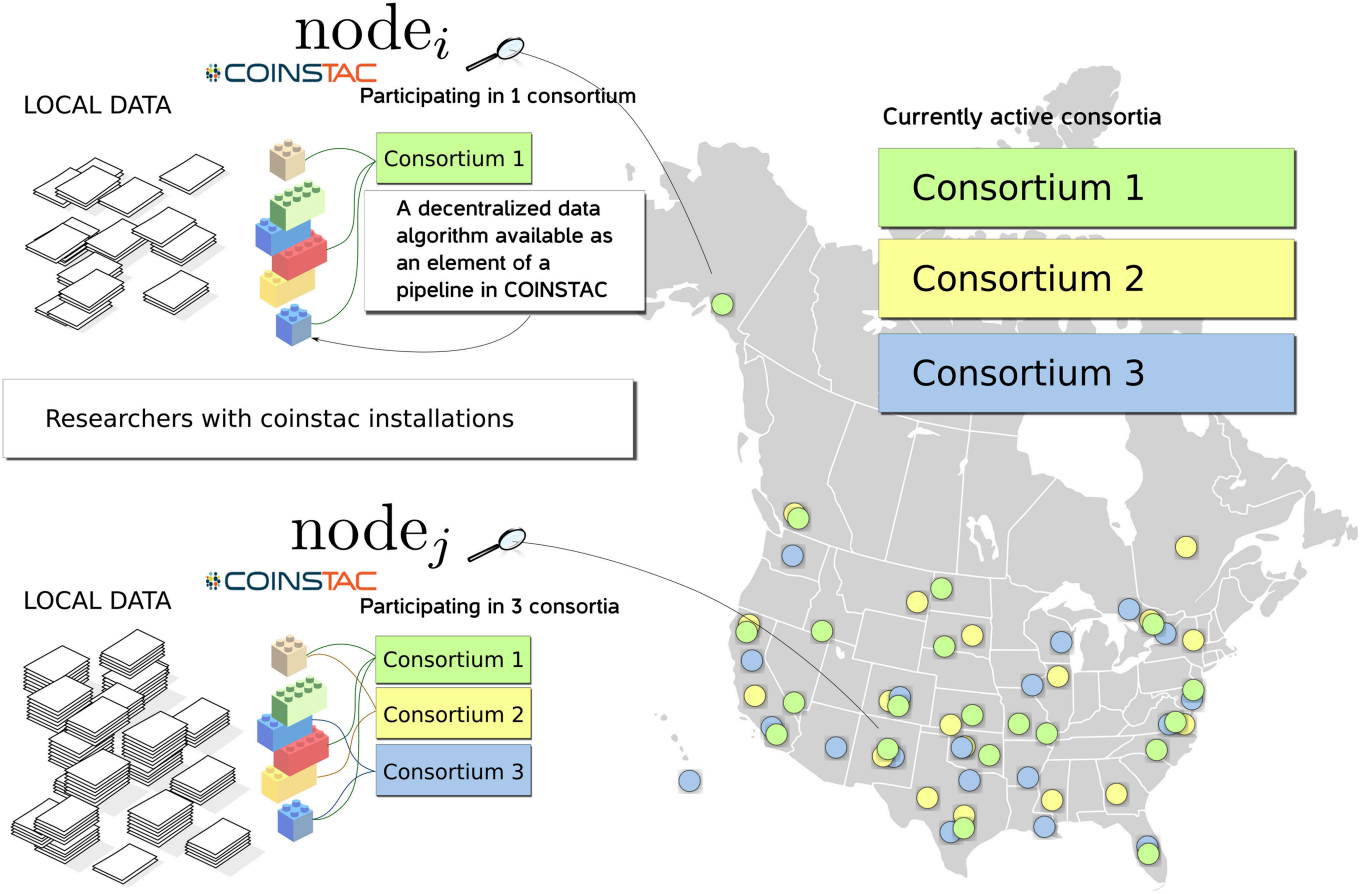
**An overview diagram of how COINSTAC organizes research on (potentially sensitive) decentralized data**.

COINSTAC is designed with the intent of providing a convenient and powerful web-based workbench for executing decentralized and privacy-preserving algorithms on locally stored data spread across organizations, and returning results to the requestor (see Figure 1). The COINSTAC system enables the same analyses that can be accomplished by aggregating shared data from multiple sites without exposing collaborators to the administrative headaches associated with actually sharing the data. In this paper we demonstrate an initial implementation of the system that implements the message passing infrastructure and includes an example subset of processing algorithms. Our primary objective is to demonstrate the organizing principles and feasibility of the tool being developed. The ultimate goal of COINSTAC is to provide a comprehensive set of tools for collaboratively processing decentralized data. COINSTAC enables this by providing a framework for performing large-scale decentralized analyses. It forms virtual clusters of sites that execute automatically generated computations on their local data and aggregate statistics using global inference rules. This communal organization provides flexible and intelligent resource management, in which a continuous stream of large scale projects can be formed and completed without the need to create new organizations or project-specific storage vaults.

### 2.1. COINSTAC model

COINSTAC will provide users a venue for widespread sharing and aggregate analyses of multimodal datasets, while maintaining granular control of privacy. Building upon the existing capabilities of COINS (Scott et al., 2011; Landis et al., 2016), this next-generation system inherits powerful data collection, integration and management capabilities, which have been “battle-tested” with more than 40,000 imaging datasets and other data-types (http://coins.mrn.org). COINSTAC will enable users to move from data acquisition to sharing with minimal effort. To demonstrate this model, the COINSTAC prototype has easy-to-use protocols for attaching new sites to the system. We provide a uniform data interface (UDI) to allow data sources to maintain local file organization, without having to make separate copies for using COINSTAC.

COINSTAC model is based on the following key components:

**Ease of Interaction and Collaboration:** The purpose of the COINSTAC interface is to actively promote collaborations and data sharing. The current prototype is a platform for building and running new algorithms that operate on decentralized datasets. However, these algorithms when fully interfaced to preprocessing pipelines, pipelined themselves, and augmented with our planned key solutions will provide a number of important functions. Users will be able to easily search for relevant data in COINSTAC, as well as invite new users or data contributions to the system. Whether working collaboratively or individually, users will be able to form virtual clusters of data visible to them, on the fly, while generating relative meta-statistics or comparison benchmarks of their own local data. Although these features are not yet fully implemented in the prototype they represent a significant part of the motivation for building it.
**Decentralized Algorithms:** COINSTAC implements a highly flexible approach to pipelining complex data analysis algorithms (PCA, ICA, NMF, regression) within a chosen collaboration by enabling decentralized version of the many commonly used processing methods. This work is ongoing: we have developed, used and published decentralized methods for regression, PCA, NMF, and ICA which will be added to our prototype in the near future (Potluru et al., 2014; Baker et al., 2015; Imtiaz et al., 2016). In sections below we explain the details of their operational backbone—the decentralized gradient descent—and show how common analyses such as regression can be realized within such a framework.
**Privacy Preserving Data Mining:** To expand the reach of the system to data sets which may be impossible to share, COINSTAC will incorporate “differential privacy” to most of the algorithms made available for decentralized processing^6^, which enables privacy management at a granular level. We already have this implemented within our prototype for the regression analyses. The approach that we will explain below will allow us to add privacy guarantees to many algorithms that use decentralized gradient descent for the solution. Furthermore, in COINSTAC contributing sites control privacy permissions for their data.
**Web-based End-user Platform:** COINSTAC uses a web-based platform for end-users to interact with clients/nodes. This will make the platform available and scalable to as many clients and users as needed. The web-based tools allow local administrators to configure and curate locally stored data and user accounts. More broadly accessible tools allow querying nodes on their data, their computational capabilities, and the nature of virtual consortia in which they may wish to participate.

**Figure 2:**
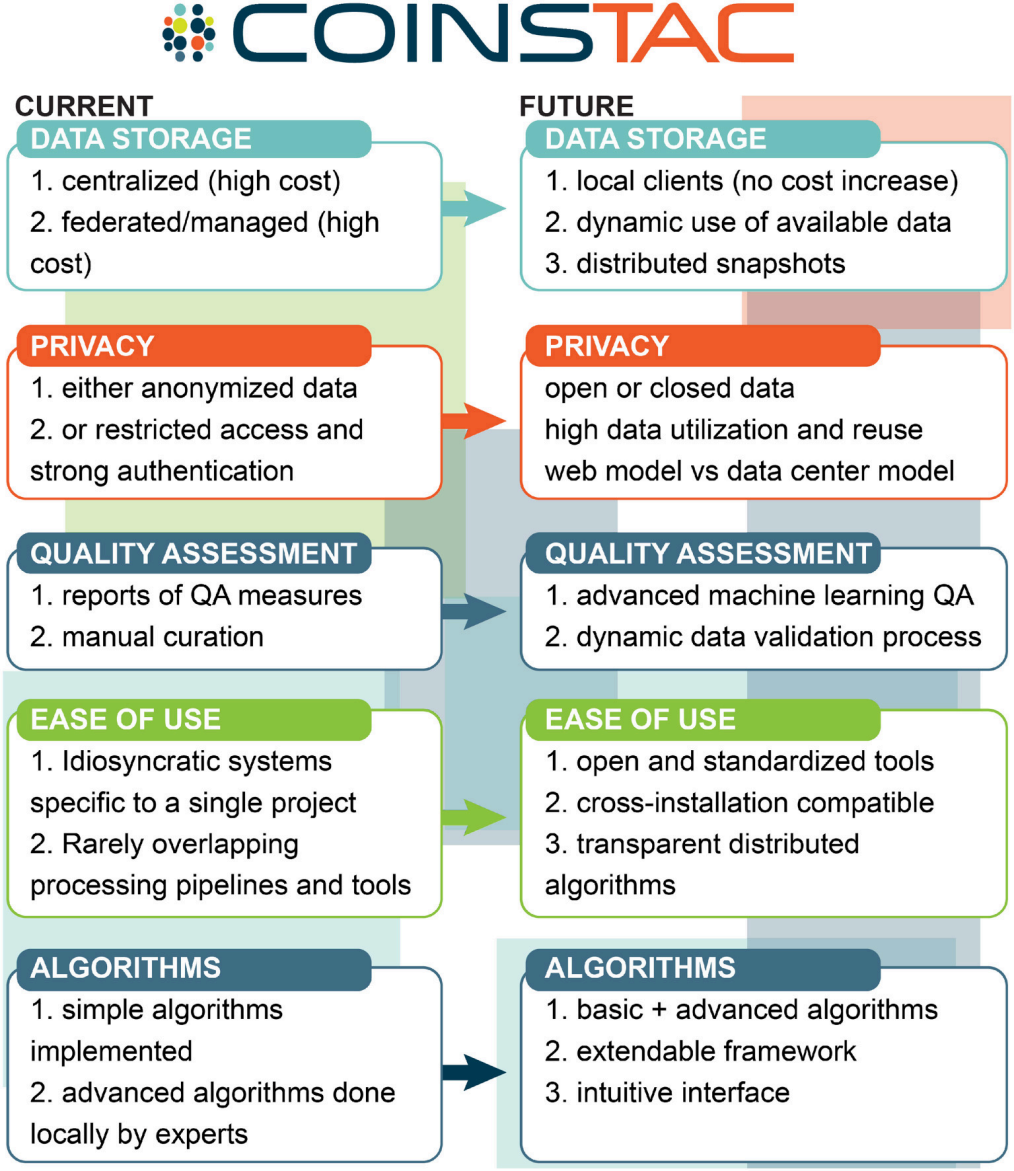
**COINSTAC vision for multisite data analysis relative to the current most common approaches**.

The overall vision of how we see COINSTAC pushing state-of-the-art multisite data analysis is summarized in Figure 2.

### 2.2. Algorithms for decentralized data analysis

Our automated system for analysis of decentralized datasets immensely simplifies existing collaboration methods. We make a distinction between single-shot schemes, in which local sites compute some summaries of their local data which are then combined by a central aggregator, vs. iterative methods, in which the aggregator and local sites communicate back and forth to refine their results. Existing systems such as ENIGMA manually support single-shot analyses; our goal in COINSTAC is to automate this using software (scripts) that can enable a larger number of participating sites with minimal delays. Our infrastructure also allows us to implement iterative analyses on a large number of sites, something which is virtually impossible using the manual approach due to the overhead of delays and human stress. We illustrate the approach on a linear regression problem.

Consider a setting where there are *N* sites and each site *j* has a data set D_*j*_ = {(**x**_*i, j*_, *y*_*j*_):*i* ∈ {1, 2, …, *n*_*j*_}} where ${\text{x}}_{i,j}\in {\mathbb{R}}^{d}$ is a *d*-dimensional vector of real-valued features, and *y*_*j*_ ∈ ℝ is a response. That is, each site has *n*_*j*_ data points for a linear regression. The goal is to fit a linear model that relates the features to the responses:

(1) $$y\approx w^{⊤}x+b.$$

It is standard to include the offset *b* as part of **w**:

(2) $$w\leftarrow \left[\begin{array}{c} w\\ b \end{array}\right]$$

(3) $$x\leftarrow \left[\begin{array}{c} x\\ 1 \end{array}\right].$$

Thus from now on we will consider learning in the model

(4) $$y\approx w^{⊤}x.$$

A popular way of implementing linear regression in this setting is *ridge regression* (Hastie et al., 2009), in which the vector of regression coefficients is found by minimizing the following function:

(5) $$F_{j}(w)=\sum_{i\;=\;1}^{n_{j}}{\left(y_{i}-w^{⊤}x_{i,j}\right)}^{2}+\frac{\lambda }{2}\;∥w{∥}^{2}.$$

This function measures a weighted combination of the total squared-error from predicting *y*_*i*_ using ${\text{w}}^{⊤}{\text{x}}_{i,j}$ and the squared length of **w**. The $\frac{\lambda }{2}||\text{w}|{|}^{2}$ term, known as a regularizer, helps prevent overfitting.

To get an objective function across all of the nodes, we can just try to minimize the sum of their local objectives:

(6) $$F(w)=\sum_{j\;=\;1}^{N}F_{j}(w).$$

An objective function in this form is called separable (since it splits over the sites), and such functions have been studied extensively in distributed optimization, signal processing, and machine learning. We assume that a central aggregator node (which we call Agg) would like to compute the minimizer of *F*(**w**).

#### 2.2.1. Single-shot analysis

In a single-shot approach (like that of ENIGMA), each site *j* can find the minimizer ${\hat{\text{w}}}_{j}$ of its own local objective *F*_*j*_(**w**), thereby solving the local regression problem. They would then send these to Agg who could average the vectors $\left\{{\hat{\text{w}}}_{j}:j=1,2,\ldots ,N\right\}$ or perform a more sophisticated meta-analysis. Minimizing *F*_*j*_(**w**) can be done with standard off-the-shelf solvers included in most programming libraries. The pseudocode is shown in Algorithm 1.

**Algorithm 1:** Single-shot averaging

**Require:** Data D_*j*_ at site *j* for sites *j* = 1, 2, …, *N*, where |D_*j*_| = *n* for all *j*.
1: **for** *j* = 1 to *N* **do**
2: ${\hat{\text{w}}}_{j}={\text{argmin}}_{\text{w}}F_{j}\left(\text{w}\right)$.
3: Node *j* sends ${\hat{\text{w}}}_{j}$ to Agg.
4: **end for**
5: Agg computes $\hat{\text{w}}=\frac{1}{N}\overset{N}{\underset{j=1}{\sum }}{\hat{\text{w}}}_{j}$ return $\hat{\text{w}}$

Averaging is the simplest aggregation method, but more sophisticated strategies may yield better results. For example, our earlier work demonstrated a data-aided aggregation strategy that treats local sites as providing new features for the aggregator (Sarwate et al., 2014), which provides significant improvements over averaging.

In addition to regression, we can use the single-shot framework to measure the average value of some measure across the datasets at different sites. For example, averaging the volume of the hippocampus across all sites from healthy adults gives some idea of the expected value of a hippocampal volume in the normal population. This assessment includes many sources of variability: imaging parameters and contrasts, different methods for measuring the volume, and precision in extracting volume estimates. A more interesting example is the effect size for disease, such as the difference in hippocampal volume between individuals with long term schizophrenia and individuals without psychosis. Such rapid automated assessments could be used to give a quick sense of the expected finding was well as the range of variability.

Averaging all of the predictors with equal weights has some justification statistically if each of the data sites has the same amount of data (|D_*j*_| = *n* for all *j*). If each of the sites draws its data from the same population according to the same sampling distribution, then each of the sites will have statistically identical data and should produce statistically identical regression coefficient ${\hat{w}}_{j}$. Thus averaging the coefficients is like taking the sample average from a population—the mean predictor should be a better estimate of the best predictor for the population. For smaller sample sizes, we have shown that a data-aided aggregation strategy that treats local sites as providing new features for the aggregator (Sarwate et al., 2014) provides significant improvements over averaging: this works if Agg has its own data set.

A major limitation of single-shot averaging is when data sets at different sites have widely varying sizes. This is even more complex if the sites contain different populations. A variable weighting scheme could recover some performance (Sarwate et al., 2014), but there are no “canonical” solutions to addressing this variability. One approach is through ensemble learning techniques (Dietterich, 2000; Rokach, 2010; Mendes-Moreira et al., 2012), which have been used to aggregating classifiers and regressors in a data-driven manner.

#### 2.2.2. Iterative analysis

Rather than using an off-the-shelf optimization method locally (as in Algorithm 1) we can use an iterative approach. This entails implementing an optimization method in a distributed fashion by having the sites and Agg communicate iteratively. This is a form of distributed gradient descent. The gradient of *F*(**w**) is the sum of the gradients of the local functions:

(7) $$\nabla F(w)=\sum_{j\;=\;1}^{N}\nabla F_{j}(w),$$

where

(8) $$\nabla F_{j}(w)=-2\sum_{i\;=\;1}^{n_{j}}(y_{i}-w^{⊤}x_{i,j})x_{i,j}+\lambda w.$$

At each time *t*, the algorithm has Agg send the value of the regression coefficients **w**_*t*_ to each of the nodes, which then compute their local gradients ∇*F*_*j*_(**w**_*t*_) and send them back to Agg. The aggregator Agg takes the average of the gradients and takes a gradient step.

Algorithm 2 shows the basic decentralized gradient descent procedure. The algorithm passes the iterate to the local sites which return gradients and the local objective value. If the objective increased, the algorithm backtracks and halves the step size, otherwise it continues descending along the gradient. For the regression objective we can use the gradient formula above, but for more complex objectives we may have to approximate the gradient. An alternative approach to using a tolerance check on the gradient is to run the procedure for a fixed number of iterations: such differences in implementation may depend on the particular data set being analyzed.

**Algorithm 2:** Decentralized gradient descent

**Require:** Data D_*j*_ at site *j* for sites *j* = 1, 2, …, *N*, where |D_*j*_| = *n* for all *j*, step size η, **Tol** for convergence
1: Agg initializes **w**_*c* − 1_ = **0**, *F*(**w**_*c* − 1_) = +∞, ∇*F*_*c* − 1_ = 2·**Tol**·**1**
2: **while** ||∇*F*_*c* − 1_||> **Tol do**
3: **for** *j* = 1 to *N* **do**
4: Agg sends **w**_*c* − 1_ to node *j*
5: Node *j* computes ∇*F*_*j*_(**w**_*c* − 1_) and *F*_*j*_(**w**_*c* − 1_)
6: Node *j* sends ∇*F*_*j*_(**w**_*c* − 1_) and *F*_*j*_(**w**_*c* − 1_) to Agg
7: **end for**
8: Agg computes ∇*F*_*c*_ ← ∑ ∇*F*_*j*_(**w**_*c* − 1_). ⊳ aggregate gradient
9: Agg computes **w**_*c*_ ← **w**_*c* − 1_ − η∇*F*_*c*_. ⊳ gradient step
10: Agg computes $F\left({\text{w}}_{c}\right)=\overset{N}{\underset{j=1}{\sum }}F_{j}\left({\text{w}}_{c}\right)$
11: **if** *F*(**w**_*c*_) > *F*(**w**_*c* − 1_) **then**
12: η ← η∕2 ⊳ reduce step size if overshoot
13: **else**
14: Agg sets $\nabla F_{c-1}=\overset{N}{\underset{j=1}{\sum }}\nabla F_{j}\left({\text{w}}_{c}\right)$. ⊳ commit updates
15: Agg sets *F*(**w**_*c* − 1_) = *F*(**w**_*c*_).
16: Agg sets **w**_*c* − 1_ = **w**_*c*_
17: **end if**
18: **end while**
19: **return w**_*c*_

Another application in which an iterative gradient descent procedure is useful is decentralized joint ICA (djICA) (Baker et al., 2015).

**Figure 3:**
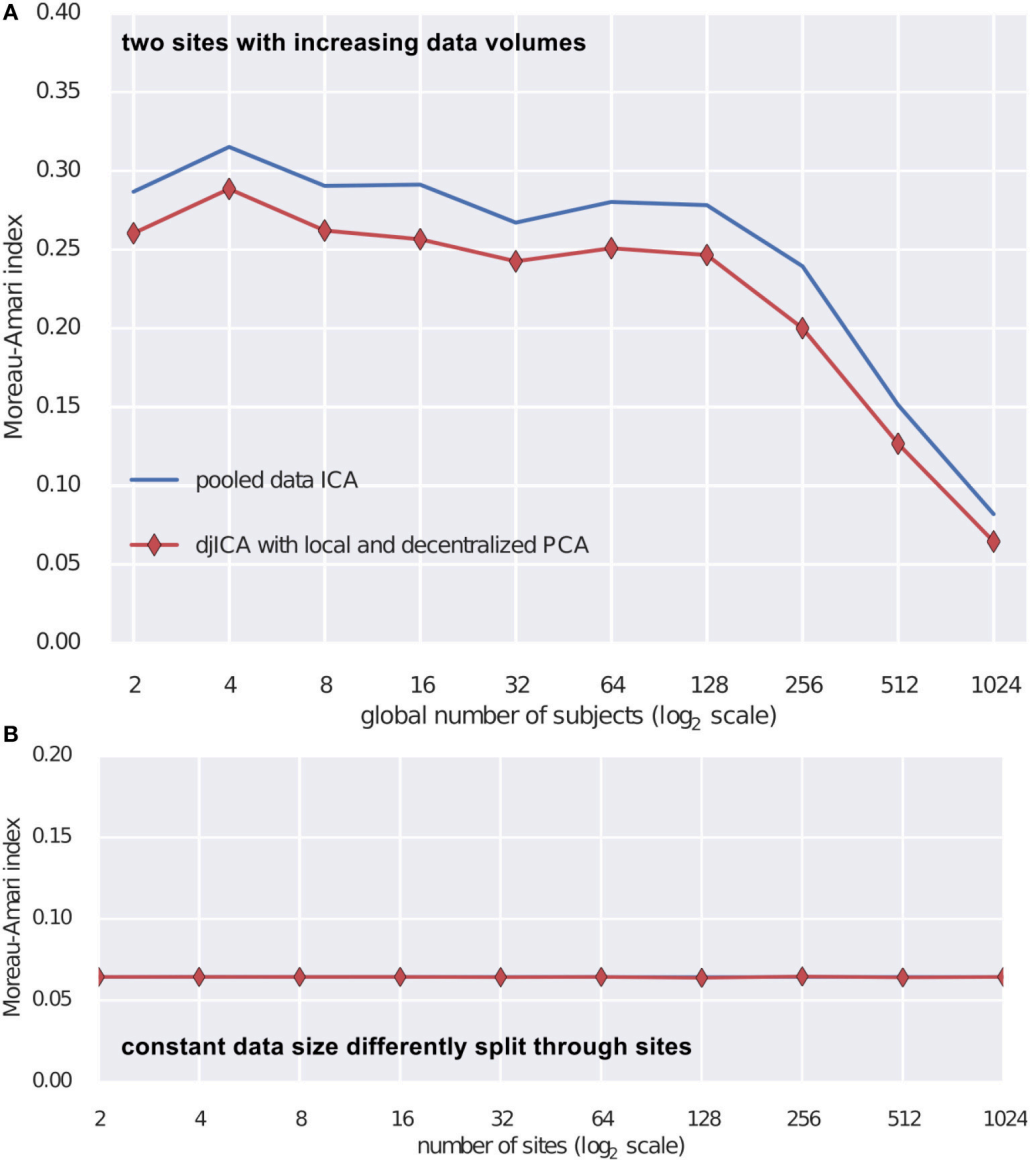
**Comparison of djICA ISI with that of pooled data ICA**. **(A)** Effect of increasing the number of subjects in an experiment with two sites. Distributed ICA has performance competitive with centralized ICA even though the data is split across different sites and only derivatives are shared. **(B)** Effect of splitting a total subject pool of 2048 subjects among an increasing number of sites. There was no visible change in performance despite the data being distributed.

To test the djICA algorithm we used the fMRI simulation toolbox simTB (Erhardt et al., 2012)^7^ to generate spatial components. Using these components we formed the ground truth mixing matrix **A**. For time series we have used a realistic model of fMRI component time series (Lindquist et al., 2014). Mixing the ground truth time courses with **A** we obtain simulated subjects. Assuming a subject per site we test djICA and compare it to performance of a single local ICA on the pooled data starting with 2 participating sites and increasing them up to 1024 (Figure 3A) and also keeping the total number of subjects at 2048 while splitting them across sites from 2 to 1024 (Figure 3B). Figure 3 summarizes performance of the algorithm by displaying Moreau-Amari index (Macchi and Moreau, 1997) that compares the quality of the estimation of the unmixing matrix against the ground truth mixing and is invariant to the scaling and permutation ambiguities of ICA. The results convincingly demonstrate that with increased sample size the quality of feature estimation increases and this is true for both pooled data ICA and our proposed djICA which performs competitively although the data is not shared between the sites. Moreover, we have found that splitting across sites does not degrade the results given the same data volume.

### 2.3. Differential privacy

COINSTAC's decentralized data analysis approach also opens the possibility of using otherwise inaccessible data. As discussed before, access to “Raw” data may not be available for many reasons (Sarwate et al., 2014). As discussed earlier, COINSTAC automates the manual aggregation approach of ENIGMA, which allows researchers to share intermediate results and data derivatives. However, some organizations or individuals may not be allowed to share direct data derivatives due to privacy concerns related to subject re-identification. One source of privacy concerns is a focus on the ethics of informed consent: subjects may have agreed to allow their data to be used for one study, but even sharing “aggregates” may not be covered by the initial consent form. Legal issues abound in data sharing, especially across state and national lines: the European Union's data privacy laws may not be compatible with the HIPAA regulations in the United States. In order to maximize the ability to access this type of data COINSTAC supports an infrastructure and algorithms that mathematically guarantee privacy of data while providing a secure technical framework to still enable efficient analysis on all these otherwise inaccessible datasets.

The key to providing these privacy guarantees is quantifying privacy risk (and a working technical platform that enables you to do something with it). Differential privacy is a recently proposed framework to accomplish this goal (Dwork et al., 2006). Notably, the notion of privacy in defined as a property of an algorithm, not a dataset. Differentially private algorithms try to control the risk that individual data points can be inferred from the output of the algorithm. This means that differentially private algorithms are randomized. For example, a differentially private regression algorithm would produce a random vector of regression coefficients. Ideally, the distribution of the output will be concentrated around good coefficients (high utility) while remaining insensitive to variations in the input that represent the presence or absence of individuals (high privacy).

To understand the privacy guarantee, consider an algorithm A which could have one of two inputs: a database D containing *n* individuals, or a nearly identical database D′ containing *n* − 1 of the individuals in D and one different individual, so that where D has *x*_*i*_, databased D′ has $x_{i}^{\prime }\neq x_{i}$. The idea is that the output A shouldn't reveal whether the *i*-th individual had *x*_*i*_ or $x_{i}^{\prime }$. For a deterministic function A (such as the average) A(D) ≠ A(D′) so this would not guarantee privacy. We say that a randomized algorithm A guarantees (ϵ, δ)-differential privacy if

(9) $$\mathbb{P}(A(D)\in \;S)\leq \exp\left(\epsilon \right)\cdot \mathbb{P}(A(D^{\prime })\in \;S)+\delta .$$

for any measurable^8^ set of outputs S ⊆ Y.

Since being proposed by Dwork et al. (2006), differentially private algorithms have been designed for a number of applications, including statistical parameter estimation (Kasiviswanathan et al., 2008; Smith, 2008, 2011; Chaudhuri and Hsu, 2011, 2012), regression and classification (Chaudhuri et al., 2011; Kifer et al., 2012; Rubinstein et al., 2012; Yuan et al., 2012; Song et al., 2013; Bassily et al., 2014; Jain and Thakurta, 2014; Sheffet, 2015). An exhaustive description of these works is beyond the scope of the current paper—we direct the interested reader to the recent monograph of Dwork and Roth (2014) and the tutorial by Sarwate and Chaudhuri (2013).

In the context of distributed learning, some general algorithms have been proposed that work on the principle of averaging the outputs of computations from local data (Wang et al., 2013; Huang et al., 2014; Ji et al., 2014; Song et al., 2015); a recent study by the authors found that differentially private aggregation of local classification rules could potentially lead to major improvements in overall accuracy (Sarwate et al., 2014). Theoretical guarantees for distributed optimization algorithms are often asymptotic and do not give reasonable predictions for the smaller sample sizes typically encountered in neuroimaging applications.

We can adapt both our single-shot and iterative approaches to make the procedures differentially private. For the single-shot case, local sites can replace their local algorithm with a differentially private counterpart. In an iterative computation model, the sites can send noisy gradient evaluations. In general the noise distribution will depend on many application-specific parameters: the privacy risk ϵ, the dimension of the data, the range of data values, the specific objective function being optimized, and so on.

#### 2.3.1. Single-shot approaches

The simplest approach to aggregation is in a single-shot. In this approach, COINSTAC receives a request from a researcher and generates sharing requests to the data holders. The data holders automatically runs a differentially private computation on their local data and share the result to the researcher who can then aggregate the results. As a simple example, consider computing an average: there are *N* sites *j* = 1, 2, …, *N*, each of which has *m* different samples {*x*_*i, j*_:*j* = 1, 2, …, *n*}. The goal is to estimate the mean across all of the data, which is assumed to follow a simple model:

(10) $$x_{i,j}=\mu +z_{i,j}$$

where *z*_*i, j*_ is standard normal N(0, 1) measurement noise for the *i*-th sample at site *j*. As described in our earlier work (Sarwate et al., 2014), if the actual data is bounded so *x*_*i, j*_ ∈ [0, 1] each data holder can share a differentially private version of their local means:

(11) $${\tilde{X}}_{i}=\frac{1}{m}\sum_{j\;=\;1}^{m}x_{i,j}+\frac{1}{\epsilon m}w_{i},$$

where *w*_*i*_ is a Laplace random variable with unit variance. The researcher can then average these noisy averages to form an estimate μ with variance $\frac{1}{Nn}+\frac{1}{{\left(\epsilon n\right)}^{2}N}$.

The single-shot approach can also be used for regression or other optimization-based problems, as shown in Algorithm 3: a site *j* with data pairs {(*x*_*i, j*_, *y*_*i, j*_)} of variables and effect can compute a differentially private local estimate of regression coefficients ${\tilde{\text{w}}}_{j}$ using any ϵ-differentially private method A(D, ϵ). They can send these to an aggregator which will then aggregate the vectors. A simple approach is to estimate by the average: $\hat{w}=\frac{1}{N}\sum_{i=1}^{N}{\tilde{w}}_{i}$. However, using more advanced ensemble learning techniques (Dietterich, 2000; Rokach, 2010; Mendes-Moreira et al., 2012) can result in better aggregation. If the researcher has their own data set, they can also use it for better ensemble learning (Sarwate et al., 2014).

**Algorithm 3:** Differentially private single-shot averaging

**Require:** Data D_*j*_ at site *j* for sites *j* = 1, 2, …, *N*, where |D_*j*_| = *n* for all *j*, privacy parameter ϵ, differentially private optimization method A(D, ϵ).
1: **for** *j* = 1 to *N* **do**
2: ${\tilde{\text{w}}}_{j}=A\left(D_{j},\epsilon \right)$.
3: Node *j* sends ${\tilde{\text{w}}}_{j}$ to Agg.
4: **end for**
5: Agg computes $\tilde{\text{w}}=\frac{1}{N}\overset{N}{\underset{j=1}{\sum }}{\tilde{\text{w}}}_{j}$ return $\tilde{\text{w}}$

#### 2.3.2. Iterative approaches

Single-shot communication settings work for problems in which the goal is aggregation, but do not allow for feedback and refinement of the results. We can also implement differentially private iterative algorithms for learning from decentralized data. The challenge is that every time the local sites transmit information about their data they “leak” information about their private data. Under the standard differential privacy calculus, the total privacy risk is the sum of the risks from these sequential disclosures. In order to protect against this, each iteration has to be itself more private (hence more noisy), leading to a tradeoff between privacy and utility in terms of the number of iterations. In essence, a single analysis can come with a total *privacy budget*: the challenge is how to spend that budget across iterations of the algorithm. This tradeoff appears in centralized analyses as well. For example, the private power method of Hardt (2013) uses an iterative procedure to compute a singular value decomposition (SVD) for principal component analysis (PCA). For regression and classification, differentially private stochastic gradient methods (Song et al., 2015) can be used to process large data sets to iteratively improve the regression coefficients. Such noisy iterative procedures may suffer from increased variability: techniques such as minibatching can often help recover performance in the face of additional noise (Song et al., 2013).

There are two ways to implement differentially private iterative methods within COINSTAC. The simplest is to use the stochastic gradient approach outlined in Algorithm 4, which is a variant of stochastic gradient descent (SGD). We replace the sites' gradient with a differentially private counterparts. However, because the returned values are noisy, the algorithm will not have monotonically improving performance (Song et al., 2015). Another approach would be to cycle sequentially through the sites, as in djICA (Baker et al., 2015): each site could optimize/update (in a differentially private manner) the iterate **w**_*t*_ and then pass it to another site. By cycling through the sites repeatedly, this approach could refine the iterate while satisfying an overall privacy budget.

**Algorithm 4:** Differentially private stochastic gradient descent

**Require:** Data D_*j*_ at site *j* for sites *j* = 1, 2, …, *N*, where |D_*j*_| = *n* for all *j*, time *T*, learning rate parameter η, privacy parameter ϵ, regularization parameter λ
1: Agg sets **w**_1_ = **0**
2: **for** *t* = 1 to *T* **do**
3: **for** *j* = 1 to *N* **do**
4: Agg sends **w**_*t*_ to node *j*
5: Node *j* generates privacy-preserving noise **Z**_*j*_.
6: Node *j* computes **v**_*j, t*_ = ∇*F*_*j*_(**w**_*t*_) + **Z**_*j*_.
7: Node *j* sends **v**_*j, t*_ to Agg.
8: **end for**
9: Agg computes ${\text{w}}_{t+1}={\text{w}}_{t}-\frac{1}{\lambda t}\overset{N}{\underset{j=1}{\sum }}{\text{v}}_{j,t}$
10: **end for**
11: **return w**_*T* + 1_

### 2.4. Web-based platform

One of the main aims of our implementation of the decentralized data analysis system is to maximize democratization of the discovery process and allow as many users (individual researchers and research groups) to form consortia and perform data analysis and metadata exchange regardless of their location and constraints. However, it is clear that some requirements have to be set for participation. Availability of network connection and computational resources (i.e., a workstation) are something that cannot be avoided. Our goal however is to minimize the requirements. Although it may seem logical to require a consortium researcher to bring in data to the virtual team to participate in a project, it is also true that allowing researchers without data may also be of benefit. Similarly, in ENIGMA people contribute their expertise even when they have no data, however, this process is not automated. It may be of value for some consortia to have an option of adding subcontractors, remote participants or crowd-sourcing the effort of optimal pipelining of the data pre-processing. Forcing members to contribute data may limit the researchers who are able to participate in a virtual project. Addressing the requirement of democratization of research, we have made two key decisions: (i) to develop the client and the server based on web technologies for client-server interactions, and (ii) to transfer data through the HTTP protocol. The first decision led to an extremely portable tool that is able to run on any of the three major operating systems. While the second requirement greatly increases the user-base as it enables the clients to function without any additional special permissions from local IT departments. Essentially this is similar as accessing a web-page with the same risks but with all the additional benefits of COINSTAC.

**Figure 4:**
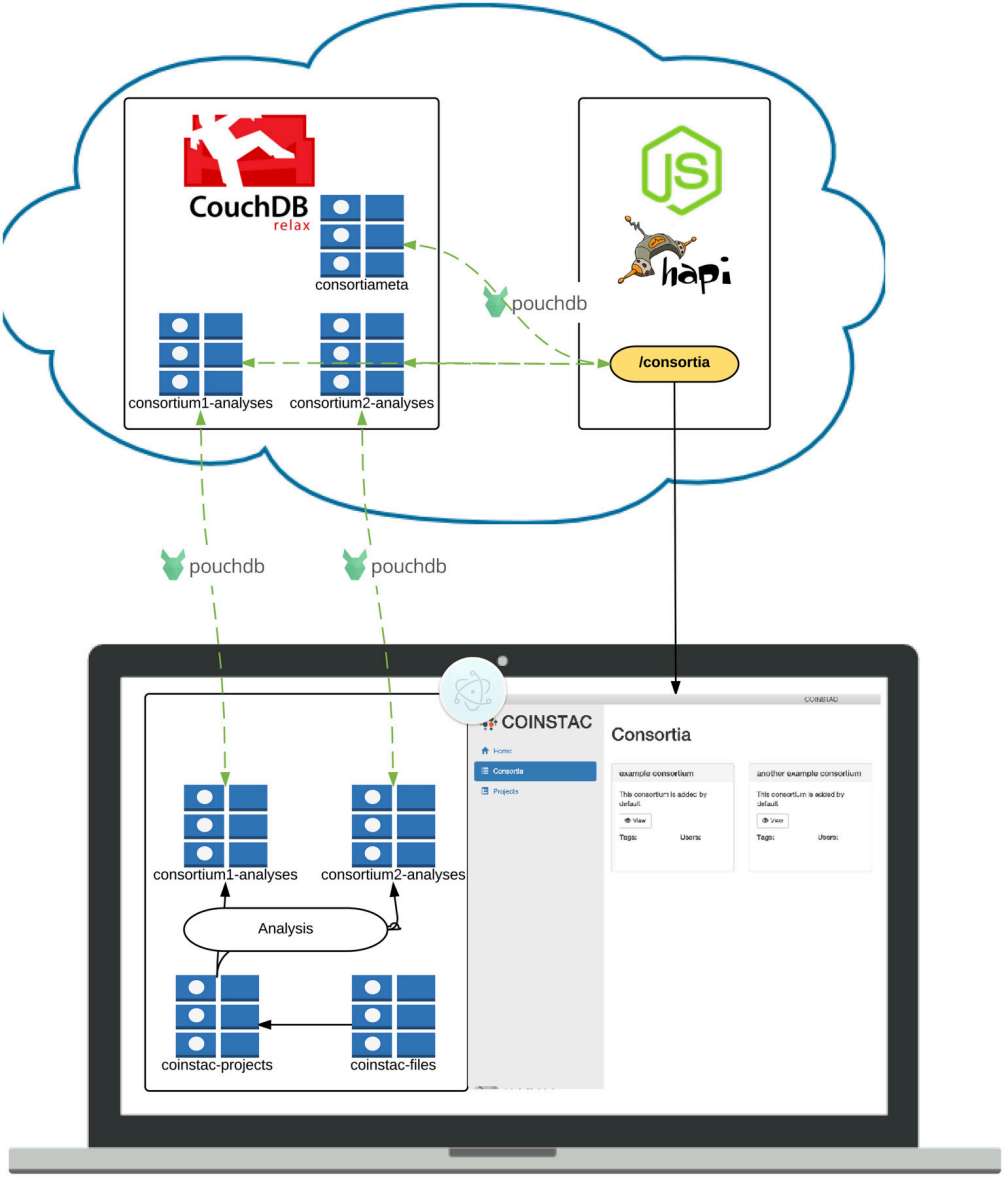
**Architecture of the COINSTAC implementation**. The client is a desktop application written using standard web technologies on top of the Electron (2015) and Node.js (2015). Electron enables installation on any major OS and access to low level system utilities not available to browser-based web apps. The server is a RESTful API running on top of Node.js (2015) and Hapi.js (2015). Data is stored in multiple CouchDB (2015) datastores. Client-server data transfer takes place via the RESTful webservice, and synchronization mechanisms that are built into CouchDB (2015).

We have create a software implementation guided by these main requirements with the objective of further democratizing research and enabling decentralized consortia and projects. The overview of its architecture is listed in Figure 4; we describe some of the system components, design choices, and technical details of this implementation in the remainder of this section.

The COINSTAC application uses standardized web communication protocols to synchronize (meta)data between each client and web-wide accessible synchronization node that maintains consortia-specific data stores. Data stores are noSQL document stores managed by CouchDB (2015). CouchDB exposes a RESTful interface through which data can be retrieved and modified. CouchDB also provides mechanisms for eventually consistent replication, which allows multiple replicas of the same database to receive both read and write requests simultaneously. While database storage is provided by CouchDB, the syncronization part is provided by PouchDB JavaScript library.

The cloud-based datastores are complemented by a web server, which controls access to data that should not be visible to all clients. The number of web service endpoints hosted by the web server is greatly reduced by allowing direct access to the CouchDB REST interfaces for each datastore. There are still some endpoints required to handle tasks such as user creation and authentication. The web server hosts a RESTful web service powered by Node.js (2015) and the Hapi.js (2015) framework. In addition to responding to HTTP requests, the Node process monitors the noSQL datastores for changes which should result in re-running aggregate analyses.

The COINSTAC client is a portable desktop application which can be installed on Windows, Mac and Linux operating systems. The application is built within Electron (2015), an open source environment for building desktop applications using web technologies. In an Electron application, a single Node.js main process starts the application, and launches a separate rendering process, which renders the user interface in a modified Webkit web browser window providing the user experience of a standalone application.

The user interface of the application is essentially a web application that interfaces with the main thread via Inter-Process-Communication (IPC) and remote APIs over HTTP. The application relies on unidirectional data flow inspired by Facebook's Flux (2015) architecture. The idea behind (Flux, 2015) is a way to make actions predictable and application state easier to reason about. Our particular implementation of this paradigm (Redux, 2015) allows for highly reasonable application state management through functional programming paradigms. This has many benefits, including hot code swaps during development^9^, predictable interaction flow and easy debugging. User interface data schemas are enforced using (Ampersand, 2015), which allow for straightforward validation and management. The interface itself is made with React (a declarative view layer); it promotes code reusable components and supporting code organization.

All of these tools—CouchDB, Electron, Redux, React, Ampersand Models, etc.—are open source and freely available to use.

#### 2.4.1. User experience

**Figure 5:**
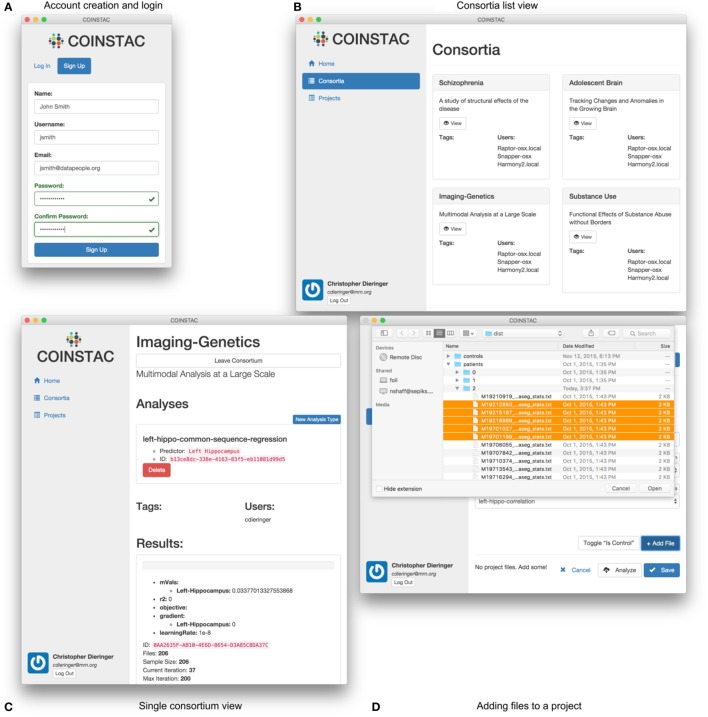
**User interface examples and parts of the COINSTAC workflow: (A) login to the system or account creation, (B) viewing (after creation) of the list of consortia the user is involved in, (C) view of a single consortium, and (D) adding files to a project within a consortium**. Consortia list in **(B)** demonstrates a case of exercising a granular control: parts (or all) of a local data pool can be involved in different consortia (studies). At the same time, a data provider only allows their data to be used for the agreed-upon purposes of a consortium; to use these data for something else, a different consortium needs to be joined. The individual consortium view in **(C)** can show progress of ongoing computations and other monitoring information. Despite the client being essentially a web application, in COINSTAC it is straightforward to add data to a consortium using a simple point and click operation via standard OS tools with which the users are already familiar.

The COINSTAC workflow is designed to be simple and intuitive. For data contributors, it consists of the following steps:

1. registration and login (e.g., Figure 5A),
2. joining a consortium (e.g., Figures 5B,C),
3. importing data (e.g., Figure 5D),
4. running an analysis, and
5. reviewing results.

Figure 5 shows the look-and-feel of the COINSTAC client prototype for some of these operations. The consortia list in Figure 5B demonstrates a case of exercising a granular control: parts of a local data pool can be involved in different consortia (studies). At the same time, a data provider only allows their data to be used for the agreed-upon purposes of a consortium; to use these data for something else, a different consortium needs to be joined. Notably, the same dataset can simultaneously be a part of multiple consortia with their specific allowed uses. The individual consortium view in Figure 5C can show progress of ongoing computations and other monitoring information. Despite, the client being essentially a web application, in COINSTAC it is trivial to add data to a consortium by a simple point and click operation via standard OS tools which users are already familiar with (Figure 5D).

**Figure 6:**
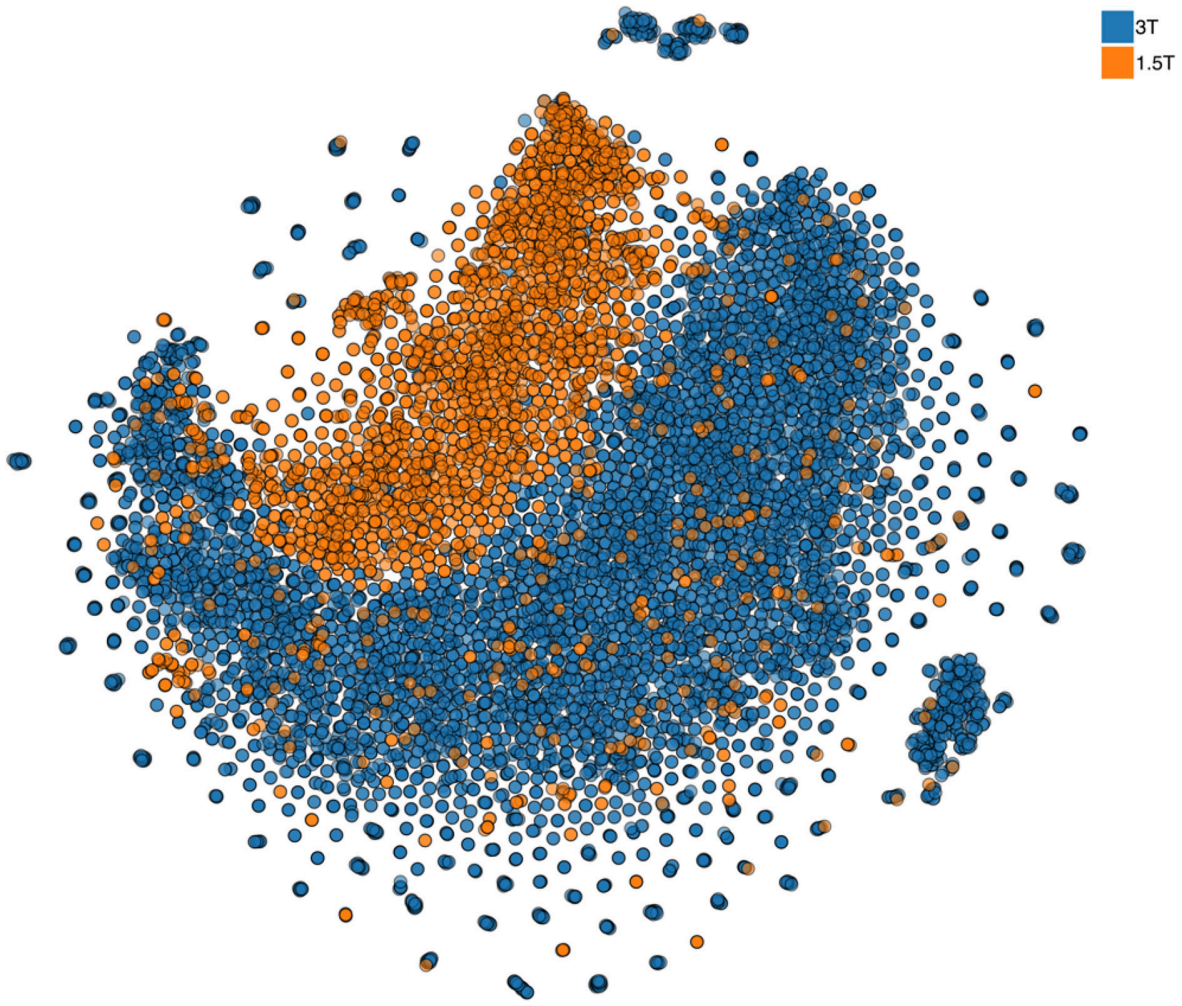
**Nonlinear 2D embedding using t-SNE on a dataset of 10,000 structural MRI scans collected at the Mind Research Network (stored in COINS)**. The colors are added at the post-processing stage and signify two different field strengths (i.e., two scanners) used for data collection. Notably, the data are visibly different for the two scanners. We also show that despite this, the coding of effects such as age is remarkably similar in the two scanners, providing support for cross-scanner studies which incorporate calibration factors (Panta et al., 2016).

Continuous granular control of one's data is a core motivational component of our approach. Leaving this control in the hands of each researcher and providing consortia building tools allows us to let researchers build trustworthy collaboration, where the questions of control and access of the data are resolved prior to engagement. In part, we plan to facilitate such an approach by providing all local pre-processing tools (building on the existing code base of already used open-source software). However, approaches that simultaneously view all multidimensional data samples as points^10^ on a 2D map, similar to the one shown in Figure 6, can provide a quick overview of the complete dataset and point to inconsistencies or anomalies (see more details in Panta et al., 2016). The goal of these approaches^11^ is to represent a dataset consisting of large number of multidimensional samples in a way that preserves structure that relates the samples to each other. Similar samples thus appear as points located close together while dissimilar samples are placed farther apart. This is a helpful property for anomaly detection. If all but one sample are similar finding that odd sample may require looking at each of them in turn. With an embedding approach the odd sample will conveniently be placed apart from the rest and thus easily identified. Indeed, the method used to produce such an embedding (not unlike other methods in this class) is inherently local and relies on computation that involves computing a distance metric between each and every sample. The limitations of the current methods, however, do not at all mean that COINSTAC cannot implement a distributed nonlinear embedding schema with privacy. We already are working on an approach that can rely on a public dataset available to all researchers (as we have pointed above there are multiple open neuroimaging databases). Using this dataset as a reference a full 2D map of all data in a consortium can be constructed and used to convey the overall structure (Globerson et al., 2007) of the data with the same privacy guarantees as other decentralized data methods in the COINSTAC.

The prototype is already available but, as a prototype, it may require technical skills to install, mainly because of the need for previously described dependencies. However, the framework and approach were chosen to quickly lead to the following desirable (and already implemented) outcome. To use the system, users will first download the COINSTAC client. No installation process will be required^12^. The user, double clicks the application and begins immediate use. Users may register from within the COINSTAC login screen: an account is needed for authentication and subsequent control of consortia membership.

After an account is created and the user has logged in, he/she may navigate to the “Consortia” page, accessible from the navigation bar. Here, all consortia that are active for the current user are listed together with the high-level metadata regarding each of them. Selecting a consortium brings the user to the consortium overview page. A user can see the consortium's description, members, analyses definitions, and latest analyses aggregate results.

When the user is ready to contribute data to one of the analyses established by one of their consortium, they may visit the “Projects” page. The user may easily create a new “Project” and add files to it^13^. Once files have been added, the user may select a consortium and and analysis to run against the project's files. As each file is analyzed, the results are incrementally fired off in the background. The application will emit a notification when all files are analyzed. The application will also emit a notification when the central server has completed its aggregation, and the aggregation result is available for view in the consortium's overview page.

The web technologies and the client provide an implementation of the infrastructure that allows creation of virtual research centers (consortia) and implements all the steps of information exchange among the members of a consortium. This is a framework for decentralized data processing and information exchange. However to be useful, it needs algorithms that are able to function in a decentralized environment and analyze the data without the need to ever move it into a central place or to load all the data into the same machine RAM.

## 3. Results

The COINSTAC prototype implementation is equipped with all required infrastructure to support the algorithms of Section 2.2. The current (initial) version provides the means for communication between the master and the client nodes as well as the hooks to modify local and global gradient and objective functions. To demonstrate the COINSTAC infrastructure, we have implemented the single-shot and iterative versions of ridge regression^14^. Note, the single-shot and iterative schedules already implemented by the framework are open-ended skeletons for implementation of various decentralized data algorithms. Although several algorithms with required properties already exist, including our own djICA and multilevel classification results (Sarwate et al., 2014; Baker et al., 2015), more development is needed to support many of the popular tools used for neuroimaging analysis. There are many existing distributed algorithms which could also be adapted to decentralized data settings.

We applied two versions of ridge regression to brain structure collected as part of two different studies, one including chronic schizophrenia and healthy controls and another including substance users and healthy controls. For the first study, we used three neuroimaging datasets physically located on three different machines communicating through the HTTP protocol. The total number of subjects on all machines was 224. Each machine with the respective dataset constituted a “site,” and had roughly half (patients/controls: 35/39, 36/40, 35/39) the sample as individuals with schizophrenia and half healthy controls. Each site's data had already been analyzed using FreeSurfer 5.3 (this and other pre-processing steps will be incorporated within COINSTAC in future versions). Note, this analysis is done per subject and no cross-site communication is required at this pre-processing step although some analysis may require pipelining of decentralized operation. The COINSTAC system was used to generate the results of ridge regression regressing the right hippocampal volume on the cases and controls label. The datasets during the computation never left the sites and the volumes were computed prior to the computation at each site individually; at each site, the appropriate datasets for cases and controls were identified automatically by the local COINSTAC platform, the right hippocampal volumes for each were extracted from the original FreeSurfer output files.

For single-shot ridge regression COINSTAC follows Algorithm 1. Each site computes a local ridge regression results—beta-weights—which are communicated to the central analyzer. In our test case the betas are a single element vector (we used centered data and labels). The central analyzer averaged the results according to line 5 or Algorithm 1. The different sites were balanced for cases and controls and roughly age-matched. As expected, the right hippocampal volume was significantly reduced in individuals with schizophrenia (for the “single-shot” β = −0.00043, the fit to the pooled data was *R*^2^ = 0.061375, *p* = 1.8568*e* − 04).

For comparison purposes we also aggregated the original data and performed a regression on the full dataset; the *R*^2^ value for right hippocampal volume was very similar to (but not exactly the same as) to the distributed measure (*R*^2^ = 0.061542, *p* = 1.8185*e* − 04), as might be expected by other meta- vs. mega-analysis comparisons (Turner et al., 2013; van Erp et al., 2014).

Following the same setup of three data sites as in the case of the single-shot analysis we also ran the COINSTAC implementation of the iterative version of ridge-regression using Algorithm 2. The *R*^2^ value produced for this analysis was 0.061530. Results, even for this small example, show a clear progression of accuracy when the centralized data result is taken for the “best” value: $\frac{\text{single-shot iterative centralized}}{0.061375\;0.061530\;0.061542}$. Differences between the three approaches are clear but relatively small in this particular, relatively simple, example, but can in theory have a significant impact on the results for larger and more complex analyses as are typically run for ENIGMA. For the second study, we used the same setup, and the number of subjects for each group was the same (we used a subset of data from an existing ongoing study). We also applied the iterative (multi-shot) approach to to compare total gray matter volume in substance users vs. healthy controls (100 subjects in each group). Results showed β = −0.0000027 and show an expected statistically significant reduction in gray matter volume.

## 4. Discussion

In the current form COINSTAC is envisioned as a way for researchers with the data to quickly form consortia to test out ideas, and produce scientific results without needing to go through any data-related agreement paperwork, or infrastructural challenges of pulling all participating data together^15^. The advantage is an obvious speed up in the research operation and ability to focus on research rather than bearing the maintenance cost.

Consequently, COINSTAC is not (primarily) a system for distributing raw data among anyone who desires to conduct research with it. In this sense the system is not competing with, but rather augments the current centralized systems (Van Horn et al., 2001; Marcus et al., 2007; Dinov et al., 2010; Poline et al., 2012; Mennes et al., 2013; Poldrack et al., 2013; Autism Brain Imaging Data Exchange, 2016). COINSTAC solves problems that are not otherwise addressed: quick engagement with the data that is hard to get access to due to the paperwork—and a resulting time delay—burden, and accessing datasets that due to privacy reasons cannot be shared. As such, COINSTAC is a powerful tool for distributed research consortia at all investigation stages while their project is ongoing and before the data is fully sanitized, scrutinized and ready to be put to a central open place for anyone to work on. Thus, while the highly valuable goals of central repositories are to empower further scientific research on previously collected data, COINSTAC is primarily an agility tool for that data at all stages of research of the groups that originally conceived of the study and conducted it prior to the open-access-ready stage. Furthermore, COINSTAC complements open data sharing initiatives with ability to perform research on data that will unlikely be ever shared mostly due to privacy and legal concerns. Indeed, existing repositories can, in principle, serve as a data source for COINSTAC consortia, thus leveraging existing available data and combining with other data not available within a given repository.

Indirect access to raw data, or a lack of it in the conventional sense, may seem as an insurmountable block for quality research when evaluated from the conventional data processing wisdom. Multiple successes of the ENIGMA project (Hilbar et al., 2013; Thompson et al., 2014, 2015; van Erp et al., 2016), however, provide a strong evidence, that conventional approach to data analysis is not the only one possible. COINSTAC enables automation of the mostly manual process of communication across sites. Automation means logging of all steps and a resulting lower costs for catching and correcting errors thanks to the ease of reruns of overall analyses within a consortia. Furthermore, the differences in the operation in decentralized settings and the centralized approach will lead to different best practices that are already emerging in other data processing fields dealing with large amounts of decentralized data. For example, an approach to the quality control that may help here is a decentralized version of the results presented in Figure 6 (Panta et al., 2016). We are working on algorithms for that but others will emerge once COINSTAC gains adoption. The quality assurance tools built for the decentralized data settings and integrated with COINSTAC will allow consortia participants to mark outlier data for exclusion from the analysis without even requiring its physical relocation or access to remote hosts.

Our prototype, which also includes a differential privacy enabled ridge regression as a special case, forms the foundation for more complex single-shot analyses, such as taking into account individual age, gender, scanning site, IQ, and other covariates that might be necessary for any statistical analysis of brain measures along the lines of the ENIGMA analyses. The automation will greatly simplify and speed up the manual work required for performing “ENIGMA-like” analysis in settings outside COINSTAC. COINSTAC automates all the manual steps that are related to algorithm execution. The scripts are automatically sent: i.e., a consortium executes its pipeline transparently to the fact that the data may be spread across sites around the globe. The scripts are now pipelines, each element of which has been already implemented and vetted (the prototype only implements ridge regression at this initial point). The consortium chains the elements in the desired order and sets parameters. If the parameters did not produce a satisfactory results, the head of the consortium may tweak them and rerun the analysis until desirable outcome is achieved. All of that happens without any explicit interactions with any of the other sites that are part of that consortium. COINSTAC takes care of all of the interactions under the hood. The aggregated results, such as regression coefficients, are automatically available to each participating site upon completion of the pipeline run. The gathering, storing and message passing (that otherwise needs to be handled by numerous emails) has by then already happened unbeknownst to the happy scientist who deeply cares about the result and COINSTAC now allows them to focus on it.

Furthermore, the ability to rerun the analysis quickly and without extra overhead may be a game changer as it allows quick re-analyses often needed due to random mistakes, the need to probe parameter ranges and hyperparameter optimization. However, as it has been previously observed (Sarwate et al., 2014) the single-shot schema is guaranteed to lead to improvements only in the limit of infinite data and may not produce a desirable result if the datasets at the local site are quite different. On the other hand, the algorithms that are allowed to iterate through the sites multiple times theoretically produce the same answer as the aggregate analysis would on the same data. Empirical studies also confirm that (Potluru et al., 2014; Baker et al., 2015). These are the iterative multi-shot analysis that are heavily based on the single-shot developments algorithmically and in terms of the required infrastructure. Manually realizing iterative algorithms in decentralized data settings is impractical and COINSTAC may be one of the first neuroimaging tools allowing this option.

However, as the vision described in this paper may have indicated, individuals without data would not be able to take part in such consortia directly via installation of the COINSTAC software. This does not mean such researchers cannot participate intellectually at the pipeline planning and decision making steps—they will just not need to install COINSTAC clients as they do not have data to run the pipeline on. The key point, is that only sites that host parts of decentralized dataset need to install the tool. We can easily envision, that if the members of a consortium would like to share raw data when needed (if all the paperwork is in place), then dataless consortium members can gain access to the data. Although in this raw data sharing case the system looks rather similarly to the centralized repositories, the decentralized nature can still provide advantage of redundancy and peer-to-peer sharing to lower the networks load and increase the throughput. We have not yet actively worked on this option, but that could be a nice addition.

The current COINSTAC prototype runs on all major desktop OSs and is being designed to be available on any device; the designed responsive user interface is able to scale and adapt to many screen sizes which will be instrumental when ported to mobile platforms. CouchDB also provides mechanisms for eventually consistent replication, which allows multiple replicas of the same database to receive both read and write requests simultaneously. This feature will eventually enable hierarchical organization of multiple synchronization nodes.

Neuroimaging research is being performed on various platforms under varying (constantly updated) OS versions and using different versions of software tools. Mostly, the publications mention the software versions that were used in presented analyses but rarely every version detail of all pieces of the software complex (OS, libraries, etc.) is included. Arguably, these difference may have an impact on the outcome of a study, although we would expect that these difference may average out in the decentralized data settings. The most straightforward solution of requiring everyone to use the same platforms with the same software (and possibly hardware) versions for research is impractical and outright ridiculous as it creates more problems than it solves. Our approach, instead, is to log all of the processing and computation steps performed in a study. Having COINSTAC tool operating at all member sites enables effortless provenance of complex data processing streams and further enables us to generalize results to multiple systems. In the plans is to add features that enable extracting all of the provenance information to supplement a publication.

The algorithm schemas presented in this paper assume computation at every node is finished before an answer is generated or an iteration can proceed. Unfortunately, in a heterogeneous environment on the network we cannot expect that every node is always available. To cop with that we plan to implement multiple strategies for the algorithms to follow and for a specific consortium members to choose from. It is straightforward to enrich COINSTAC with exception handling mechanisms that inform the central computation node of any problems at the local node. This would ease the control of the process. One option is to declare every site's result is important and if errors or timeouts are happening to any of the nodes the system will escalate the issue to the operator level and would not proceed until it is fixed. When the number of sites is large, consortia members may desire to trade the desire to include each and every local node's results into the analysis for the speed of obtaining the result even on the partial data. In this case, a timeout at the master node would solve the problem. The timeout rule would also work if some of the sites has a different frequency of updates than any other. Notably, all computational and network issues are easy to track and analyze as this process will happen automatically on all clients and each site can obtain an overall dashboard with the health status of the system. These features are future work.

COINSTAC is an infrastructure tool that enables the research on decentralized data that we envision as a currently missing piece in the options available to neuroimaging researchers. The vision that entails its use is broader in many senses but an important piece we have not discussed and not covered by COINSTAC is consortia building infrastructure. COINSTAC picks up at the stage when researchers have already found each other and agreed on collaborating. We would like to work on tools that simplify and enrich this process allowing researchers to search through already established consortia, to evaluate their data against already available on the network and more. Of course, all contingent on the level of privacy and access existing consortia have allowed. One part of that system could be a statistic portal that shows usage and other statistics. This is all future work while the are working on preparing the presented prototype for wide adoption. Current use was limited to our institution running on multiple machines and processing several hundred datasets.

## 5. Conclusions and future work

In summary, we present a new concept for decentralized, privacy-enabled, processing of brain imaging data, which we believe is an important addition to the data sharing arsenal in this age of big data. We implement and demonstrate a working prototype (demonstration) called COINSTAC, and perform several “proof of concept” analyses on volumetric MRI data. Key benefits of our proposed approach (not necessarily implemented in the limited prototype, among others, include: (1) computation on decentralized data without large bandwith requirements (implemented in the prototype), (2) ability to form consortia and rapidly rerun multiple analyses with minimal effort (implemented in the prototype), (3) an option to use iterative approaches such a multi-shot regression, or more complex multivariate approaches like ICA or NMF (partially implemented in the prototype), (4) privacy enabled computation which provides quantifiable privacy protection through addition of specific noise distributions (not in the prototype yet), and (5) a focus on ease of use, enabling a simple mapping of data on one's computer to an existing file structure (not fully in the prototype). In the future, we plan substantial extensions to the existing prototype including the addition of data pre-processing and quality control functionality as well as the addition of tools for increasingly complex modeling and additional privacy-enabled algorithms. One special direction is enhanced consortia building functionality. Currently COINSTAC is just a tool that connects researchers in a project but only after they have established an agreement outside of the system. Like many existing social network tools it relies on participating researchers knowing who they want to connect with in a consortium. We envision that with the help of automated data exploration tools and some parts from the social network tools, COINSTAC may help people to meaningfully form consortia from within the environment reducing the load on researchers in establishing everything up-front. It is our hope that this approach will open up access to many data sets that currently cannot be utilized due to practical or regulatory limitations. Our general approach is of course applicable to any type of data in concept. As the size of available data continues to grow, approaches like those in COINSTAC will be in even greater demand if we want to fully leverage existing data to maximize the power of our scientific inquiry.

## Author contributions

SMP suggested the idea of the decentralized data system, layed out the initial system's architecture, developed algorithms, wrote initial paper draft and coordinated writing. AS developed algorithms with differential privacy, worked on writing the paper. DW coordinated the implementation of the prototype, including writing corresponding sections of the paper. CD developed and refined the architectural details of implementation, worked on implementation of the prototype. DL helped with architectural choices and took an active part in implementation of the prototype. CR was essential in architectural choices, infrastructure maintenance and software development of the prototype. SP provided brain imaging data processing pipelines and visualization algorithms development. JT provided global connections to the neuroimaging community, datasets maintenance and manuscript writing. JS helped with overseeing the ethical component of developing a platform for private data analysis and manuscript writing. KC provided context of development of tools in the similar idea space and worked on writing. PT helped in concept development, bringing in experience from practical use of a distributed system, writing the manuscript. KH provided substance abuse dataset and the expertise in the field. VC headed the overall project, formed the vision and architecture of the developed system, managed the team, worked on writing.

### Conflict of interest statement

The authors declare that the research was conducted in the absence of any commercial or financial relationships that could be construed as a potential conflict of interest.

## Footnotes

^1^Mainly due to removing the requirements of (a) having large file-servers capable of storing all of the data in the pool, and (b) releasing participating local data to the public.

^2^Indeed, keeping both data and computation within the cloud can effectively solve the bandwidth and local resources problem once the data have been transferred. Alas, the aforementioned ethical, legal and access restriction issues remain unaddressed.

^3^The ENIGMA team has also performed mega-analysis on a sub-set of cases, though this requires sharing of the full data to a central location and due to privacy concerns does not include genome wide analysis studies (GWAS). They are also actively working on multi-shot approaches, further demonstrating the need for such tools.

^4^We will highlight some of these approaches in this paper.

^5^These algorithm run on data spread across research centers but produce a result as if the data were in one place (e.g., Bai et al., 2005; Baker et al., 2015).

^6^The goal is to eventually provide a private version of each algorithm in the pipeline.

^7^http://mialab.mrn.org/software

^8^This is a mathematical technicality.

^9^This allows continuous development and modifications without requiring full system restart and maintaining the uptime.

^10^Note that in these approaches a multidimensional data sample is turned into a zero-dimensional point, thus plausibly preserving the privacy of that sample by collapsing all information to a much sparser representation.

^11^Usually called nonlinear dimensionality embedding.

^12^The prototype needs a presence of Node.js (2015) and its package manager pulling some dependencies into a local directory, which may require technical skills, however, the final version will only need a download.

^13^The current prototype is limited in assuming that the files are processed locally via common pipelines, however, the work is in progress on automating the preprocessing as well.

^14^The regularization parameter was λ = 0.7.

^15^To appreciate the difficulties imagine a consortia with up to a hundred participating sites.
